# Trajectories of negative and positive experiences of caregiving for older adults with severe dementia: application of group-based multi-trajectory modelling

**DOI:** 10.1186/s12877-024-04777-w

**Published:** 2024-02-19

**Authors:** Chetna Malhotra, Isha Chaudhry, Shimoni Urvish Shah, Truls Østbye, Rahul Malhotra

**Affiliations:** 1 Lien Centre for Palliative Care, Duke-NUS Medical School, 8 College Road, Singapore, 169857 Singapore; 2 Program in Health Services and Systems Research, Duke-NUS Medical School, Singapore, Singapore; 3 Centre for Ageing Research and Education, Duke-NUS Medical School, Singapore, Singapore

**Keywords:** Trajectories, Dementia, Older adults, Caregiver

## Abstract

**Background:**

Family caregivers of older adults with severe dementia have negative and positive experiences over the course of caregiving. We aimed to delineate joint trajectories (patterns over time) for negative and positive experiences, identify risk factors associated with membership of joint trajectories, and ascertain the association between joint trajectories and caregivers’ outcomes after the death of the older adult.

**Methods:**

Two hundred fifteen family caregivers of older adults with severe dementia in Singapore were surveyed every 4 months for 2 years, and 6 months after the death of the older adult. Using group-based multi trajectory modelling, we delineated joint trajectories for positive (Gain in Alzheimer Care Instrument) and negative (sub-scales of modified Caregiver Reaction Assessment) experiences of caregiving.

**Results:**

We identified four joint trajectories – “very high positive, low negative” (23% of caregivers), “high positive, moderate negative” (28%), “very high positive, moderate negative” (28%), and “high positive, high negative” (21%). Caregivers of older adults with more behavioural symptoms, and who did not receive strong emotional support from family were more likely to have “high positive, moderate negative” or “very high positive, moderate negative” trajectory. Compared to caregivers with “very high positive, low negative” trajectory, caregivers with “very high positive, moderate negative” or “high positive, high negative” trajectories expressed greater grief and distress, with the latter also having lower spiritual well-being and quality of life at 6 months after the death of the older adult.

**Conclusion:**

The caregiving experiences for older adults with severe dementia vary between caregivers but remain stable over time. Modifiable risk factors identified for trajectories involving negative experiences of caregiving may be targeted in future interventions to improve the experience of caregiving and caregiver quality of life and distress after the death of the older adult.

**Trial registration:**

http://www.clinicaltrials.gov (NCT03382223).

**Supplementary Information:**

The online version contains supplementary material available at 10.1186/s12877-024-04777-w.

## Background

Dementia is a public health priority affecting more than 55 million people worldwide [1]. Globally, it is one of the major causes of disability and dependency among older adults and is the seventh leading cause of death [2]. Community-dwelling older adults with severe dementia (henceforth referred to as older adults in this manuscript) are highly dependent on their family caregivers [3]. These family caregivers not only report negative experiences of caregiving including adverse impacts on their schedule, health, and finances [4–6], but also positive experiences of caregiving such as personal growth, gain in relationships, and spiritual growth as a result of caregiving [7, 8]. It is thus imperative that they be jointly examined to fully understand the holistic experience of caregiving for older adults, and guide interventions to improve the caregiving experience.

A plethora of literature exists regarding the experiences of caregivers of older adults, however with limitations. First, most of these studies are cross-sectional surveys [5, 6, 9, 10] or one-time qualitative interviews [11–13]. Given the progressive nature of severe dementia [9, 14], longitudinal rather than cross-sectional studies can help understand the dynamic nature of the caregiving experience. Second, some longitudinal studies conducted with caregivers of older adults have described an “average” trajectory for either the ‘negative’ [15–17], or the ‘positive’ caregiving experience [15, 16]. There has also been an assessment of heterogeneity between caregivers in their trajectories of negative experiences of caregiving [11, 14, 18], but scant literature exists in the context of heterogeneous trajectories for positive experiences of caregiving [19, 20]. Third, most of these studies have focused on persons with mild or moderate dementia, with either no or limited numbers of caregivers of older adults with severe dementia [14, 21]. A focus on older adults with severe dementia is relevant given that their caregivers will have more negative experiences of caregiving [14, 22]. Last, most existing studies have considered negative and positive experiences of caregiving separately. A joint longitudinal assessment of heterogeneity in trajectories for negative and positive experiences of caregiving is yet to be conducted, despite the evidence that such experiences co-exist throughout caregiving. A previous study conducted by members of our team among caregivers of patients with metastatic cancer delineated the trajectories for negative and positive experiences of caregiving and found that, though these trajectories varied among caregivers, they remained largely constant with time [20].

One approach to jointly assess the trajectories for negative and positive experiences of caregiving is the group-based multi-trajectory model (GBMTM) [23]. GBMTM is a statistical approach for finite mixture modelling, i.e., it is designed to identify a finite number of groups of individuals following similar trajectories over time, and allows the analysis of the interrelationship of multiple indicators for an outcome of interest - in this case, the negative and positive experiences of caregiving [24].

Existing literature provides some indication of sub-groups of caregivers at risk of experiencing higher levels of negative and positive experiences of caregiving. Caregivers of older adults with functional and cognitive limitations [25–27], behavioural and neuropsychiatric symptoms [14, 26, 28, 29], lack of ability to communicate [30], and those with low socio-economic status [29], greater financial burden [31] or provide longer hours on caregiving [32] report more negative experiences of caregiving. Furthermore, caregivers co-residing with older adults report both negative and positive experiences of caregiving [10, 31]. Although there is little empirical literature linking resilience with negative and positive experiences of caregiving, some qualitative studies suggest that more resilient caregivers are likely to experience positive emotions during caregiving, to consider caregiving as part of their duty, and to maintain their social relationships despite their caregiving activities [33]. On the other hand, caregivers receiving additional support in caregiving activities and having a strong family network have lower levels of negative but higher levels of positive experiences of caregiving [25, 31].

Experiences of caregiving also influence caregivers’ outcomes after the death of the older adult. When death is preceded by a prolonged and intense period of caregiving, as is the case in dementia caregiving, many caregivers experience an end to the stressors of caregiving and may report improved psychosocial outcomes (relief or stress reduction theory) after the death of the older adult [34]. As caregiving burden ends, caregivers may experience relief and they may be able to pursue their previously neglected social and employment roles, self-care, and leisure activities, thus allowing them to adjust better to a life without the older adult [35, 36]. In contrast, the resource depletion theory suggests that some caregivers may instead fare worse after the death of the older adult [37]. Exposure to caregiving stress over a long period of time may diminish caregivers’ coping resources over time, and their ability to have a sense of closure following older adults’ death. When coping resources are diminished, caregivers may have difficulties adjusting during bereavement, resulting in distress and complicated grief [35]. The association between negative experiences of caregiving and caregivers’ outcomes after the death of the older adult thus remains unclear. Literature also shows that caregivers reporting higher levels of positive caregiving experiences may have worse outcomes after the death of the older adult [34, 38]. These caregivers may be deriving a sense of satisfaction from their caregiving experience and the loss of this role may lead to a loss of purpose for them. However, most studies are limited to examining caregivers’ grief and psychological distress after the death of the older adult [34, 37, 38]; overall quality of life and spiritual well-being, have been less examined [39, 40]. None have examined the relationship between *trajectories* of negative and positive experiences of caregiving and caregivers’ outcomes after the death of the older adult. Greater clarity regarding this relationship would enable early identification of caregivers likely to experience worse outcomes after the death of the older adult.

To address these gaps, our *first aim* was to use GBMTM to delineate joint trajectories for negative and positive experiences of caregiving in a sample of family caregivers of community-dwelling older adults with severe dementia in Singapore. Our *second aim* was to identify the risk factors associated with membership of each joint trajectory. We hypothesized (H1) that caregivers of older adults with more behavioural symptoms, and functional and cognitive impairment will be more likely to follow joint trajectories representing higher levels of negative and lower levels of positive experiences of caregiving. We also hypothesized (H2) that caregivers co-residing with older adults and paying for older adults’ treatments will be more likely to follow joint trajectories representing higher levels of negative and positive experiences of caregiving; while those receiving caregiving help from a migrant domestic worker (live-in, full-time domestic workers, mostly women from neighbouring low-income countries), receiving emotional support from family, having greater resilience and being spousal caregivers will be more likely to follow joint trajectories representing lower levels of negative and higher levels of positive experiences of caregiving. Our *third aim* was to assess the association between the joint trajectories and caregivers’ outcomes six months after the death of the older adult. We hypothesized (H3) that caregivers following joint trajectories representing higher levels of negative and positive caregiving experience would report worse outcomes after the death of the older adult including higher grief and distress, and poorer overall quality of life and spiritual well-being.

## Methods

### Setting

This study was conducted in Singapore, a Southeast Asian country where dementia affects 10% of the older population [41], most of whom are cared for at home by family caregivers [12, 42, 43]. With a rapidly ageing population [44] and declining old-age support ratio [45], dementia caregiving is an important public health issue, like elsewhere, in Singapore.

### Study design and participants

We used data from “Panel study Investigating Status of Cognitively impaired Elderly in Singapore (PISCES)” study, a prospective cohort of 215 primary family caregivers of community-dwelling older adults *with severe dementia* in Singapore. Details of the study (trial registration: NCT03382223) are published elsewhere [46]. Briefly, eligible participants were recruited from seven major public restructured hospitals, six home care foundations, and two hospices (May 2018 - March 2021). Healthcare providers at each site identified eligible participants for the study. Eligibility criteria for older adults included those with a diagnosis of dementia and Functional Assessment Staging Test (FAST) criteria 6C or higher [47]. FAST stage 6C or higher indicates older adults’ inability to handle mechanics of toileting (6C), urinary incontinence (6D), faecal incontinence (6E), say six intelligible words on an average day (7A), one intelligible word on an average day (7B), walk without personal assistance (7C) and sit without assistance or smile or hold up head independently (7D/E/F). FAST 6C to 6E represents moderately severe dementia and FAST 7 and above represent severe dementia [47]. Eligibility criteria for caregivers included age ≥21 years, being a family member and primary decision-maker for older adults’ treatment or responsible for ensuring their well-being, meeting the older adult at least one day per week, and having intact cognition as determined through Abbreviated Mental Test [48] for those aged ≥65 years. Participants were surveyed every 4 months, until the older adults’ death. Surveys were administered in the caregivers’ preferred language (English/ Mandarin/ Malay) using Qualtrics. Informed consent was obtained from all the caregivers. This study is based on data from the baseline to the 24-month follow-up survey (May 2018 to Dec 2022; up to 7 surveys per participant). The study was approved by the Institutional Review Boards at SingHealth and the National University of Singapore.

### Trajectory indicators

#### Positive caregiving experience

It was assessed using Gain in Alzheimer Care Instrument (GAIN), a 10-item measure developed and validated in Singapore [49], to assess the positive feelings and outcomes from caregiving. An example item is, “providing care to (older adult) has helped to increase my patience and be a more understanding person”. Each item was rated on a 5-point Likert scale (disagree a lot (=0) to agree a lot (=4)). The total score ranged from 0 to 40; a higher score indicated a more positive experience of caregiving.

#### Negative caregiving experience

Three subscales of the modified Caregiver Reaction Assessment scale (mCRA) were used to assess how caring for the older adult had interfered with various aspects of caregivers’ life - impact on schedule and health (8 items), impact on finances (2 items), and lack of family support (5 items). An example item is, “My health has gotten worse since I've been caring for (older adult).” The mCRA has been validated in Singapore [50]. Each item was scored on a 5-point Likert-type scale (strongly disagree (= 1) to strongly agree (= 5)). Only observations with more than half of the number of items completed in each subscale were included for analysis. The 15 items were averaged to generate a total score ranging from 1 to 5; a higher score indicated a more negative experience of caregiving.

#### Caregiver outcomes after the death of the older adult

Six months after the older adult’s death, we assessed caregivers’ grief, psychological distress, spiritual well-being, and overall quality of life. We assessed grief using Brief Grief Questionnaire (BGQ) [51, 52], to assess how much grief interferes with caregiver’s life. It included five questions. An example question is, “how much does grief still interfere with your life?”. Response options included not at all (=0), somewhat (=1), a lot (=2). Response from each question was summed to create a total score of 0 to 10, a higher score indicating higher likelihood of developing complicated grief.

We measured psychological distress using the Hospital Anxiety and Depression Scale (HADS) [53], a 14-item instrument to assess caregivers’ feelings in the past week. An example item is, “I feel tense or wound up”. Each item was measured on a 4-point Likert scale with a total score ranging from 0 to 42; higher scores indicated more severe anxiety and depressive symptoms.

We measured spiritual well-being using the Functional Assessment of Chronic Illness Therapy-Spiritual Well-Being (FACIT-Sp) [54], a 12-item scale. An example item is, “I feel peaceful”. Each item was measured on a 5-point Likert scale (0 = not at all, 1 = a little bit, 2 = somewhat, 3 = quite a bit, 4= very much), with total scores ranging from 0 to 48, higher scores indicating better spiritual well-being.

Lastly, we assessed the caregivers’ overall quality of life by asking them to rate their overall quality of life in the past week on a 1 to 7 scale with 1 being ‘very poor’ to 7 being ‘excellent’.

### Independent variables

We assessed older-adult and caregiver-related baseline factors that may predict experiences of caregiving over next two years. We included the following baseline older adult-related and caregiver-related factors:

### Older adult-related factors

#### Behavioural symptoms

Older adults’ behaviours were measured using 14 items from the Cohen-Mansfield Agitation Inventory [55] to assess the frequency of agitation behaviours. An example question is, “During the past two weeks, how often was (older adult’s) curse or was verbally threatening or insulting?”. Each item was rated on a 5-point Likert scale (1=never, 2=less than once a week, 3=once or several times a week, 4=once or several times a day, 5= a few times an hour or continuous for half an hour or more). The total score was the sum of all items, ranging from 14 to 70. A higher score indicated a greater extent of behavioural symptoms.

#### Functional, pathological, and cognitive impairments

We used subscales from Bedford Alzheimer Nursing Severity Scale (BANS-S) [56] to measure functional (difficulties in dressing, eating, and mobility), pathological (sleep disturbance and muscle rigidity or contraction), and cognitive (loss of speech and eye contact) impairments. BANS-S has a total of 7 items with 4 ordered categories denoting no impairment to complete impairment. Older adults who were deemed “completely dependent” on either dressing, eating, or mobility were considered functionally impaired. Older adults who experienced frequently irregular or severely disrupted sleep, or those who experienced somewhat rigid or contracted muscles were deemed pathologically impaired. Older adults who had moderately decreased ability to speak or were mute; or those who rarely or never maintained eye contact were considered cognitively impaired.

### Caregiver-related factors

We assessed whether caregivers co-resided with older adults; time spent on caregiving each day (in hours); whether received caregiving help from a migrant domestic worker; used their Medisave (national health savings account set aside from income to pay for their own or dependent’s treatments) [57] to pay for older adults’ treatments; received emotional support from family (yes/ no); and psychological resilience. Resilience was measured using the shortened Connor-Davidson Resilience Scale [58] to assess how caregivers adapt when faced with problems. It included 2 items (ability to adapt to change and ability to bounce back after illness or hardship) measured on a 5-point Likert scale ((1= not true at all, 2 = rarely true, 3 = sometimes true, 4 = often true, 5 = true nearly all the time). Total score ranged from 0 to 10 with a higher score indicating more resilience.

#### Co-variates

Included caregivers’ ethnicity (Chinese vs non-Chinese), and relationship with the older adult (adult child vs others).

All validated scales used in the study had good internal reliability (Cronbach’s alpha ≥ 0.7).

### Statistical analysis

We used GBMTM to delineate distinct joint trajectories of positive and negative experiences of caregiving. First, we jointly specified a single trajectory for each indicator and systematically tested a series of models with an increasing number of trajectories to identify the optimal number and polynomial function for each trajectory. To identify the appropriate polynomial function, we sequentially tested (at a significance level of 5%) each polynomial function from quintic to intercept (zero-order function). The selection criteria for an optimal number of trajectories included ≥5% membership probability for each trajectory with an average posterior probability (APP) threshold of 0.7, odds of correct classification of ≥5, and Bayesian Information Criterion (BIC) closest to zero [23]. We also assessed the percentage change in BIC between the two models and selected the model with fewer trajectories if the percentage change in BIC was less than 1%. Time axis was the time from enrollment into the survey - baseline to 24 months.

Using multinomial logistic regression, we assessed the association between baseline patient- and caregiver-related factors described above and membership for the delineated joint trajectories, controlling for covariates.

Lastly, for the sub-sample of caregivers with deceased older adults, we used linear regression models to assess the relationship between joint trajectories (independent variables) and caregivers’ outcomes after the death of the older adult (grief, psychological distress, spiritual well-being, and overall quality of life). We controlled for caregivers’ ethnicity and their relationship with older adults. We used Stata 17 for analyses.

## Results

A total of 215 caregivers of older adults participated in the study. Of the 80 older adults (37%) who died within the study period, caregivers of 76 older adults (95%) were surveyed 6 months after older adults’ death.

Table 1 shows the sample characteristics measured at baseline. Older adults were aged 82.9±8.1 years, and the majority were females (77%). Among the caregivers, 83% were adult children of the older adults, nearly two-thirds (74%) co-resided with older adults, and the majority received caregiving support from a migrant domestic worker (79%). On an average 5.2±4.4 hours were spent daily on caregiving. Nearly half the caregivers (48%) used their own Medisave to pay for older adults’ treatment expenses and 58% of the caregivers reported receiving strong emotional support from their family.

**Table 1:** Sample characteristics at baseline, *n*=215

**Older adults with severe dementia**	
Age, mean(SD^a^), range (54-101)	82.9 (8.1)
Female, n(%)	166 (77.2)
Behavioral symptoms, mean(SD), range (14-54)	22.3 (8.2)
Functional impairment, yes, n(%)	174 (80.9)
Pathological impairment, yes, n(%)	139 (64.6)
Cognitive impairment, yes, n(%)	151 (70.2)
***Caregivers***	
Age, mean(SD^a^), range (21-84)	56.7 (10.1)
Female, n(%)	149 (69.3)
Chinese ethnicity, yes, n(%)	170 (79.1)
Adult child of older adult, yes, n(%)	179 (83.3)
Co-residing with older adult, yes, n(%)	160 (74.4)
Time spent on caregiving per day, mean(SD), range (0-16)	5.2 (4.4)
Received caregiving support from migrant domestic worker, yes, n(%)	170 (79.1)
Used own Medisave to pay for older adults’ treatment, yes, n(%)	103 (47.9)
Received strong emotional support from family, yes, n(%)	124 (57.7)
Resilience, mean(SD), range (2-10)	7.6 (1.7)
***Trajectory indicators***	
Positive caregiving experience, mean (SD) range(10-40)	
At baseline, *n*=215	32.9 (6.1)
At 24^th^ month, *n*=93	32.5 (6.3)
Negative Caregiving experience, mean (SD), range (1-5)	
At baseline, *n*=215	2.9 (0.8)
At 24th month, *n*=93	2.8 (0.8)

^a^standard deviation

GBMTM model was used to identify the 4-group trajectory model describing the positive and negative experiences of the caregivers. Model fit indices are provided in the supplement (Supplementary Table 1). The 5-group joint trajectory model had the lowest BIC value; however, we selected the 4-group model as the percentage change in the BIC from a 4 to 5-group model was less than 1%, therefore, a parsimonious 4-group model was preferred.

**Fig. 1:**
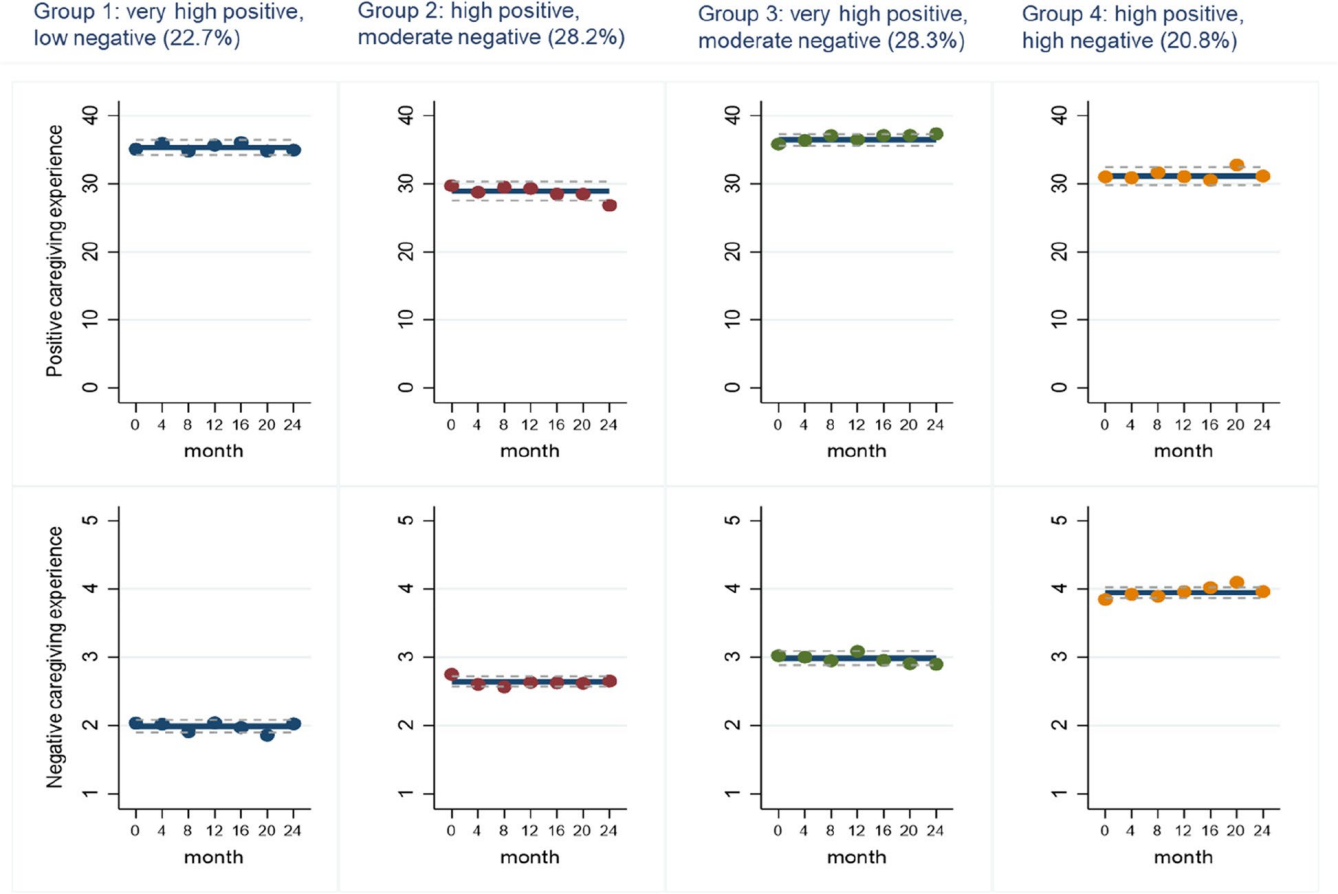
Joint trajectories of positive and negative experiences of caregiving, *n*=215

Based on the relative levels of positive and negative experiences of caregivers across the delineated four trajectories as shown in Fig. 1, we named these joint trajectories as ‘very high positive, low negative’, ‘high positive, moderate negative’, ‘very high positive, moderate negative’, and ‘high positive, high negative’ (Table 2). The trajectory ‘very high positive, low negative’ (22.7% of caregivers) had the highest positive and lowest negative experience of caregiving and was used as a reference category in our analysis. The average posterior probability of being in each trajectory was >0.8.

**Table 2:** Baseline predictors of joint trajectories of positive and negative experiences of caregiving, *N*=215

	**Joint trajectories of positive and negative experiences of caregiving** **(*ref: Very high positive, low negative*)**					
	**High positive, Moderate negative**		**Very high positive, moderate negative**		**High positive, high negative**	
	**β(SE)**	***p*-value**	**β(SE)**	***p*-value**	**β(SE)**	***p*-value**
***Older adult related factors***						
Behavioural symptoms	0.08 (0.04)	0.04	0.12 (0.04)	<0.01	0.07 (0.04)	0.06
Functional impairment, yes *(ref: no)*	1.16 (0.75)	0.12	0.49 (0.84)	0.56	1.11 (0.74)	0.13
Pathological impairment, yes *(ref: no)*	0.20 (0.54)	0.71	1.23 (0.67)	0.07	0.24 (0.54)	0.66
Cognitive impairment, yes *(ref: no)*	0.50 (0.63)	0.42	0.53 (0.71)	0.46	0.38 (0.61)	0.53
***Caregiver-related factors***						
Co-residence with older adult, yes *(ref: no)*	0.43 (0.58)	0.46	2.49 (0.89)	<0.01	0.69 (0.61)	0.26
Time spent on caregiving per day	-0.0005 (0.07)	0.99	0.19 (0.08)	0.02	0.17 (0.08)	0.03
Receives help from a migrant domestic worker, yes *(ref: no)*	-0.77 (0.84)	0.36	-1.38 (0.85)	0.10	-0.41 (0.77)	0.59
Paid for older adults’ treatment using own Medisave, yes *(ref: no)*	1.16 (0.57)	0.04	1.28 (0.64)	0.05	-0.29 (0.58)	0.61
Received strong emotional support from family, yes *(ref: no)*	-1.40 (0.61)	0.02	-3.39 (0.71)	<0.01	-1.07 (0.62)	0.09
Resilience	-0.42 (0.19)	0.02	-0.25 (0.21)	0.22	-0.15 (0.22)	0.51
Adult child of older adult, yes *(ref: no)*	0.11 (0.75)	0.88	-0.78 (0.76)	0.31	0.65 (0.71)	0.36
Chinese, yes (ref: non-Chinese)	2.13 (1.11)	0.05	0.79 (0.81)	0.33	-0.91 (0.63)	0.15

### High positive, moderate negative trajectory (28.2%)

Caregivers of older adults with more behavioural symptoms, and those who paid for older adults’ treatment expenses using their own Medisave were more likely, whereas those who received strong emotional support from their families and had greater resilience were less likely to follow this trajectory compared to those in the reference trajectory (H1, H2, Table 2).

### Very high positive, moderate negative trajectory (28.3%)

Caregivers of older adults with more behavioural symptoms, those co-residing with older adults, providing longer hours of caregiving and paying for older adults’ treatment expenses using their own Medisave were more likely, while those receiving strong emotional support from their families were less likely to follow this trajectory, compared to those in the reference trajectory (H1, H2, Table 2). The sub-group of caregivers of deceased older adults following this trajectory experienced more grief and distress 6 months after the death of the older adult compared to those in the reference trajectory (H3, Table 3).

**Table 3:** Association of joint trajectories of positive and negative caregiving experiences with caregivers’ bereavement outcomes, *N*=76

	**Grief**		**Psychological distress**		**Spiritual well-being**		**Overall quality of life**	
	**β(SE)**	***p*-value**	**β(SE)**	***p*-value**	**β(SE)**	***p*-value**	**β(SE)**	***p*-value**
Very high positive, low negative, *n*=16	*reference*							
High positive, Moderate negative, *n*=24	0.87 (0.78)	0.27	4.18 (2.17)	0.06	-5.77 (3.00)	0.06	-0.52 (0.41)	0.21
Very high positive, moderate negative, *n*=22	1.75 (0.77)	0.03	6.31 (2.15)	0.01	-5.64 (2.97)	0.06	-0.60 (0.41)	0.14
High positive, high negative, *n*=14	3.35 (0.88)	<0.01	12.04 (2.47)	<0.01	-13.30 (3.41)	<0.01	-1.38 (0.47)	<0.01

Models were controlled for caregivers’ ethnicity and their relationship with person with severe dementia

### High positive, high negative trajectory (20.8%)

Caregivers of older adults spending more time on caregiving were more likely to follow this trajectory, compared to the reference trajectory (H1, Table 2). The sub-group of caregivers of deceased older adults following this trajectory experienced greater grief and distress, and lower spiritual well-being and quality of life 6 months after the death of the older adult compared to those in the reference trajectory (H3, Table 3).

## Discussion

Using prospective data, our study is the first to assess heterogeneity in trajectories for negative and positive experiences of caregiving over 2 years among caregivers of older adults with severe dementia. We identified four joint trajectories – “very high positive, low negative”, “high positive, moderate negative”, “very high positive, moderate negative” and “high positive, high negative”.

Consistent with a previous study, we found that experiences of caregiving, both negative and positive remained stable over time [20]. It is possible that caregivers in our study sample had been caring for older adults for the past several years and since the onset of dementia. Therefore, these caregivers were unlikely to make changes in how they appraise their experience after several years of caregiving, explaining why reports of both negative and positive experiences of caregiving remained stable over time.

Notably, our results showed that although negative experiences of caregiving varied more widely among caregivers, caregivers’ positive experiences remained generally high. This may reflect the specific cultural context in which this study was conducted. In Asian cultures, adult children (constituting 83% of our sample) are bound by the tradition of filial piety and consider caring for older family members to be their responsibility [13]. As a result, despite experiencing high levels of stressors, these caregivers may derive a sense of purpose and satisfaction in caring for the older adult.

As hypothesized, caregivers of older adults with more behavioural symptoms were more likely to belong to the trajectories representing moderate negative experiences of caregiving. Behavioural symptoms may be difficult for caregivers to manage, potentially resulting in embarrassing or abusive situations [59], physical and psychological morbidity, and social isolation for the caregiver [4]. Not surprising, the literature has been largely consistent in showing that behavioural symptoms increase negative experiences of caregiving [14, 26, 28, 29].

Consistent with the previous studies [10, 31], we found that caregivers co-residing with older adults reported more negative but very high positive experiences of caregiving. Further, caregivers spending more time on caregiving reported moderate to high negative experiences of caregiving, as shown previously [32]. We also found that caregivers who paid for treatment using their savings were more likely to follow “high positive, moderate negative”, and “very high positive, moderate negative” versus “very high positive, low negative” trajectory. While being able to help older family members with dementia in receiving treatments may improve caregivers’ self-esteem and satisfaction from caregiving, given the high out-of-pocket costs in Singapore and the long treatment period for dementia, many caregivers may experience financial burden and loss of their healthcare savings, thereby reporting more negative experiences of caregiving [12].

Our findings show that caregivers with greater resilience were more likely to follow a trajectory with a higher positive and lower negative caregiving experience. Research shows that more resilient caregivers are able to adapt and recover from the physical and psychological demands of caring for older adults [60]. Studies show higher levels of resilience to be associated with the use of more positive coping strategies, greater self-efficacy, and lower burden and stress [33, 61]. Given the positive association between resilience and caregivers’ experiences, future research should aim to develop and evaluate interventions to increase caregivers’ resilience [62, 63].

Study results suggest that caregivers need both emotional and instrumental support to cope with caregiving activities. While emotional support comes from having other supportive family members, instrumental support is primarily provided by a migrant domestic worker. Having a migrant domestic worker enables caregivers to have lower negative experiences of caregiving albeit with lower positive caregiver experiences. It is remarkable that 79% of caregivers in our sample received help from a migrant domestic worker and reflects the high contribution of these workers in caregiving activities. This arrangement allows the older adults to be cared for at home while enabling the informal caregiver to pursue employment and engage in other activities.

Our results showed that compared to the very ‘high positive, low negative’, trajectory, caregivers belonging to trajectories representing more negative caregiving experiences (‘very high positive, moderate negative’, and ‘high positive, high negative’) experienced worse bereavement outcomes. These results are consistent with the ‘resource depletion theory’, which suggests that a high and sustained level of caregiver stress accumulated through negative experiences of caregiving may diminish caregivers’ coping resources over time [37]. Depletion of coping resources may leave the caregiver more vulnerable after the death of the older adult and interfere with grief resolution. Resultantly, these caregivers experience greater distress, lower spiritual well-being, and lower quality of life at 6 months after the death of the older adult. Notably, we find that these worse bereavement outcomes may happen despite caregivers experiencing high or very high positive caregiving experiences. This is consistent with previous studies suggesting that many caregivers with complicated grief report having positive caregiving experiences [38].

The main strength of our study is that it is a longitudinal prospective study with multiple assessments conducted over 2 years. Our findings add to the growing body of literature on caregivers’ negative and positive experiences, examining them jointly and longitudinally, and assessing their impact on caregivers’ outcomes after the death of the person. Our study also has limitations. Firstly, there was missing data at each time point. It is possible that missing data was not at random and may represent caregivers who did not complete the study because of older adults’ sickness or death. However, the trajectory analysis employed uses full-information maximum likelihood to handle missing data, which is more efficient than other ways of handling missing data [64–66]. Data from all caregivers regardless of whether they had missing information were used to estimate the model [67]. Secondly, the scales used to measure negative and positive experiences of caregiving do not have cut-offs for clinically meaningful values, therefore we are unable to comment on that. Thirdly, our results on the association between experiences of caregiving and caregivers’ outcomes after the death of the older adult are based on a sub-sample of caregivers whose older adult had died during the study duration. Lastly, given the unique cultural context in Singapore, the generalizability of our findings needs to be tested in different settings.

Our study results provide some indications on how to improve caregivers’ experiences. Although positive experiences of caregiving were generally high, there is potential to further improve caregivers’ sense of mastery and enable them to find meaning in their caregiver role. Training caregivers to anticipate and manage older adults’ behavioural symptoms can reduce negative experiences of caregiving. Evidence also suggests that training family caregivers to use non-pharmacologic strategies is more effective in reducing these symptoms than pharmacologic interventions [68]. Additionally, training caregivers to be resilient may prevent worse outcomes. Evidence suggests that interventions such as resilience training, cognitive behavioural therapy, mindfulness, and promoting the use of digital resilience monitoring tools in clinical settings could help strengthen the resilience and well-being of the caregivers of older adults [69, 70]. Further, social support interventions such as peer-support groups, befriending schemes, family support, and remote internet and technological support may reduce the psychological burden of the caregivers by protecting them against social isolation and loneliness experienced in the process of caregiving [71].

## Conclusion

The experience, over time, of caregiving for older adults with severe dementia is heterogenous, with varying extent of negative and positive experiences. Health and/or social service practitioners working with family caregivers of older adults with severe dementia should be mindful of this heterogeneity. Modifiable risk factors for trajectories involving negative experiences of caregiving identified in this study, like more behavioural symptoms of older adults with severe dementia, co-residence, and financial and emotional support available to caregivers, can be considered as targets in future interventions. With caregivers having a “high positive, high negative” trajectory (vs “very high positive, low negative”) expressing greater grief and distress, and lower spiritual well-being and quality of life six months after the death of the older adult, it is important to continue to care for (past) family caregivers even after the end of their caregiving role due to the death of the older adult.

### Supplementary Information

**Additional file 1:** **Supplementary Table 1.** Summary of model fit indices. **Supplementary Table 2.** Characteristics of bereaved caregivers, *n*=76.

## Data Availability

Data is available on reasonable request from the corresponding author.
