# Supplementary material for: Trajectories of negative and positive experiences of caregiving for older adults with severe dementia: application of group-based multi-trajectory modelling
Source: BMC Geriatr. 2024 Feb 19;24:172. doi: 10.1186/s12877-024-04777-w (PMC10875866; doi:10.1186/s12877-024-04777-w)
Supplement: Supplementary file 1 — Additional file 1: Supplementary Table 1. Summary of model fit indices. Supplementary Table 2. Characteristics of bereaved caregivers, n=76. [file 12877_2024_4777_MOESM1_ESM.docx]

**Supplementary Material**

**Supplementary Table 1. Summary of model fit indices**

| No. of groups | BIC | % change in BIC |
| --- | --- | --- |
| 1 | -4239.23 | - |
| 2 | -3941.31 | 7.0% |
| 3 | -3794.49 | 3.7% |
| 4 | -3728.11 | 1.7% |
| 5 | -3707.81 | 0.5% |

**Supplementary Table 2. Characteristics of bereaved caregivers, n=76**

| Grief adjustment, mean (SD^a^), range(0-9) | 3.6 (2.5) |
| --- | --- |
| Psychological distress, mean (SD), range(0-31) | 9.6 (7.5) |
| Spiritual well-being, mean (SD), range(18-56) | 42.3 (10.2) |
| Mood, mean (SD), range(0-10) | 6.2 (2.2) |
| Overall quality of life, mean(SD), range(1-7) | 5.2 (1.3) |

Note: ^a^ Standard Deviation

**PISCES Study Group members**

Chetna Malhotra^1,2^

Padmini Vishwanath^1^

Jing Rong Yong^1^

Truls Østbye^2^

Rahul Malhotra^2, 3^

Dennis Seow^4^

Phillip Yap^5^

Lay Ling Tan^6^

Weng Yew Tham^7^

Janhavi Vaingankar^8^

Jason Foo^9^

Boon Yeow Tan^10^

Kamun Tong^11^

Wai Chong Ng^12^

John Carson Allen Jr^13^

Weng Mooi Tan^14^

Shiou Liang Wee^2,15^

Li Ling Ng^6^

Richard Goveas^16^

Vanessa Mok^6^

Alisson Sim^6^

Wei Fern Ng^6^

Hon Khuan Wong^6^

Bharathi Balasundaram^6^

Rui Qi Tan^6^

Pui Sim Ong^6^

Chin Yee Cheong^5^

Alethea Yee Chung Pheng^17^

Christina Tiong^18^

Allyn Hum^19^

Angel Lee^20^

Eric A. Finkelstein^1,2^

^1^Lien Centre for Palliative Care, Duke-NUS Medical School, Singapore

^2^Program in Health Services and Systems Research, Duke-NUS Medical School, Singapore

^3^Centre for Ageing Research and Education (CARE), Duke-NUS Medical School, Singapore

^4^Department of Geriatric Medicine, Singapore General Hospital, Singapore

^5^Geriatric Centre, Khoo Teck Puat Hospital, Singapore

^6^Department of Psychological Medicine, Changi General Hospital, Singapore

^7^Care for the Elderly Foundation, Singapore

^8^Research Division, Institute of Mental Health, Singapore

^9^Alzheimer’s Disease Association, Singapore

^10^St. Luke’s Hospital, Singapore

^11^Post-acute & Continuing Care, Jurong Community Hospital, Singapore

^12^Hua Mei Centre for Successful Ageing, Tsao Foundation, Singapore

^13^Centre of Quantitative Medicine, Duke-NUS Medical School, Singapore

^14^Agency for Integrated Care, Singapore

^15^Geriatric Education and Research Institute, Alexandra Health Pte Ltd, Singapore

^16^Department of Geriatric Psychiatry, Institute of Mental Health, Singapore

^17^Assisi Hospice, Singapore

^18^Home Nursing Foundation, Singapore

^19^Dover Park Hospice, Singapore

^20^St. Andrew’s Community Hospital, Singapore
